# Concomitant Glenohumeral Instability and Rotator Cuff Injury: An Epidemiologic and Case-Control Analysis in Military Cadets

**DOI:** 10.5435/JAAOSGlobal-D-22-00049

**Published:** 2022-04-12

**Authors:** Liang Zhou, Shawn M. Gee, Matthew A. Posner, Kenneth L. Cameron

**Affiliations:** From the Department of Orthopaedic Surgery, Tripler Army Medical Center, Honolulu, HI (Dr. Zhou); the Department of Orthopaedic Surgery, Fort Belvoir Community Hospital, Fort Belvoir, VA (Dr. Gee); and the John A. Feagin Jr. Sports Medicine Fellowship, Keller Army Community Hospital, West Point, NY (Dr. Posner, and Dr. Cameron).

## Abstract

**Introduction:**

Concomitant rotator cuff tear and glenohumeral instability in a large cohort of young and active patients has not been examined. The purpose of this study was to investigate the incidence, associated variables, and outcomes in military cadets undergoing shoulder stabilization procedures with these concomitant pathologies.

**Methods:**

A retrospective cohort study of a consecutive series of collegiate patients who underwent shoulder stabilization from 2014 to 2018 at a single service academy was conducted. Exclusion criteria were noncadets, revision instability cases, multidirectional instability, and prior rotator cuff repair. A nested case-control analysis was done in a matched series of patients with and without MRI evidence of rotator cuff tear. Baseline demographics, VAS pain scale, physical therapy duration, and time to surgery were analyzed. Postoperative metrics included rate of recurrent instability, subjective outcomes, VAS pain scale, and military-specific criteria.

**Results:**

Three hundred twenty-four cadets met the inclusion criteria, including 272 men and 52 women averaging 20.53 ± 1.80 years of age. MRI demonstrated concomitant rotator cuff tears in 5.56% of cases. A matched case-control comparison between patients with (rotator cuff tear group) and without (no rotator cuff tear group) rotator cuff tear showed no differences in preoperative data, recurrent instability rate, or postoperative VAS pain scores (0.24 versus 0.88, *P* = 0.207) at mean 44-month follow-up. Fifteen of 17 patients (88.2%) in each group returned to full activity (*P* > 0.999). No patients failed to graduate due to shoulder concerns. No patients in the rotator cuff tear group underwent a medical board for separation from the military compared with 2 (11.8%) in the no rotator cuff tear group (*P* = 0.163).

**Conclusions:**

The incidence of concomitant rotator cuff tears in this study of military cadets undergoing shoulder stabilization was 5.56%. In a matched cohort comparison, the presence of a rotator cuff tear on preoperative MRI was not associated with inferior clinical outcomes.

Shoulder instability including dislocations and subluxations is endemic in young athletes and active-duty military populations.^1,2,3,4^ Anterior shoulder instability typically results from a traumatic dislocation event, and the likelihood of recurrence has an inverse correlation with patient age.^5^ Bankart lesions, which involve a tear of the anterior-inferior labrum, are the most common injury and occur in 90% of patients.^6^

Associated injuries, such as rotator cuff tears, have also been reported with acute traumatic shoulder instability events. Robinson et al^7^ demonstrated a 25.7% incidence of rotator cuff tears and greater tuberosity fractures, most commonly occurring in patients older than 60 years. Other studies have noted the prevalence of rotator cuff tears to range between 7% and 54%, with increasing frequency correlating with advancing age.^8,9^ Recognition of concomitant injury in the instability patient is critical, as its presence may present an additive barrier to surgical management and functional recovery.

A systematic review of outcomes after management of capsulolabral lesions with concomitant rotator cuff tears found improved pain relief and patient satisfaction in surgically repaired rotator cuff tears in comparison to nonsurgical management; however, most patients in the reviewed studies were older than 40 years.^10^ In a small cohort of professional rugby football players, 5/6 athletes had returned to play at 34-month follow-up after undergoing a two-stage procedure (open rotator cuff repair followed by open shoulder stabilization), and all had no recurrent instability.^11^

To our knowledge, no large studies exist on the incidence and outcomes of concomitant rotator cuff injuries in a young active-duty military population with glenohumeral instability. The purpose of this study was to investigate the incidence, associated variables, and outcomes in military cadets undergoing shoulder stabilization procedures with these concomitant pathologies. Our hypothesis was that the overall incidence of combined findings would be low and that functional outcomes would not be inferior in these patients in comparison to patients with instability alone.

## Methods

### Study Design and Setting

**Figure 1:**
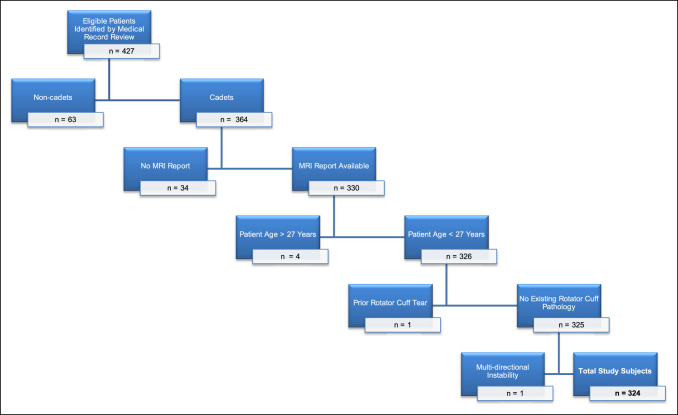
STROBE (Strengthening the Reporting of Observational Studies in Epidemiology) study flowchart. Patient exclusion criteria are as specified. MRI = magnetic resonance imaging.

This study was reviewed and approved by an institutional review board. A retrospective cohort study was conducted in a consecutive series of cadets undergoing primary shoulder stabilization surgery from 2014 to 2018 at a single military medical treatment facility (Figure 1). Study enrollment was limited to cadets at a single military service academy. The primary indication for surgery was a history of traumatic shoulder instability episode(s), given the known recurrence risk with nonsurgical management in this patient population.^12-14^ Exclusion criteria included noncadets, age >28 years, multidirectional instability, absence of an MRI report, and prior rotator cuff repair.

Clinical data were obtained from medical records and included surgical reports, outpatient clinical encounters, and relevant imaging studies to include MRI or magnetic resonance arthrogram. All imaging studies were conducted using a 1.5 Tesla magnet and read by a staff radiologist. Follow-up duration was established by the date of the most recent orthopaedic or military service physical examination.

Formal MRI reports were evaluated. When the presence of rotator cuff pathology was noted, findings were separated into discrete categories, based on the degree of tendon tear and the specific tendon(s) affected. A nested case-control analysis of arthroscopically treated patients was done, matching controls (shoulder instability alone; no rotator cuff tear [no-RCT] group) with cases (shoulder instability and concomitant rotator cuff tear; rotator cuff tear [RCT] group) based on age, sex, and date of surgery. Specified postoperative metrics were then measured within this nested analysis.

### Study Participants

The epidemiologic analysis identified 427 total patients who underwent primary shoulder stabilization surgery within the defined period. A total of 103 patients were excluded (63 noncadets, 34 with unavailable MRI reports, 4 patients aged >28 years, 1 prior rotator cuff repair, and 1 multidirectional instability), for a series total of 324 cadets (Figure 1). Preoperative data collected included age at the time of surgery, sex, procedure done, and rotator cuff status as reported by MRI. There were 272 males and 52 females in the study group, with a mean (range) age of 20.53 ± 1.80 years (17 to 28 years). Procedures done included arthroscopic labral repair in 292 cases, open Bankart repair in 6 cases, open humeral avulsion of the glenohumeral ligament repair in 2 cases, and open Bristow-Latarjet procedure in 24 cases (Table 1).

**Table 1:** Demographics and Procedures Done for Patients Who Underwent Shoulder Stabilization

Demographic Variable	Category	n	%	Mean (Range + SD)
Age (yrs)	<20	169	52	20.53 (17-28 ± 1.8)
	20-25	152	47	—
	>25	3	1	—
Sex	Men	272	84	—
	Women	52	16	—

Procedures Done	Category	n	%
Arthroscopic stabilization	Labral repair/capsulorrhaphy	292	90
Open stabilization	Bankart repair	6	2
	Humeral Avulsion of the Glenohumeral Ligament repair	2	1
	Latarjet procedure	24	7

### Evaluation of Baseline Data

A nested case-control analysis of arthroscopically treated patients with concomitant injury and those with instability pathology alone is shown in Table 2. Patients with rotator cuff tears (RCT group) on MRI report were matched with an equivalent number of patients without tears (no-RCT group) using the aforementioned criteria. Patients with <6 months of follow-up were excluded. A total of 34 patients were reviewed. Data points included age, laterality of the affected shoulder, preoperative Visual Analog Scale pain scale, follow-up duration, duration of physical therapy, time from injury to MRI, time from MRI to surgery, and collective time from injury to surgery.

**Table 2:** Matched Cohort Analysis of Patients With Rotator Cuff Tear (RCT Group) and Without Rotator Cuff Tear (No-RCT Group)

Variable	Rotator Cuff Tear Group (RCT; n = 17)	No Rotator Cuff Tear Group (No-RCT; n = 17)	*P* (of Means)
Sex, n male (%)	16 (94.12)	16 (94.12)	
Left laterality, n (%)	9 (52.94)	9 (52.94)	
Age, yrs (mean, median, range ± SD)	20.76, 21, 18-24 ± 1.75	20.18, 20, 18-23 ± 1.74	0.373
Follow-up duration, mo (mean, median, range ± SD)	43.38, 49.77, 6.57-81.60 ± 23.05	44.78, 41.93, 9.83-84.67 ± 24.31	0.878
Preoperative VAS pain scale (mean, 95% CI, range ± SD)	1.82, 0.88-2.76, 0-5 ± 1.98	1.47, 0.46–2.48, 0-6 ± 2.12	0.455
Duration of physical therapy, mo (mean, 95% CI, range ± SD)	10.35, 3.83-16.87, 0.50-40.00 ± 13.72	7.88, 4.06-11.71, 0.5-24 ± 8.04	0.464
Time from injury to surgery, mo (mean, 95% CI, range ± SD)	18.99, 7.74-30.25, 0.6-82.73 ± 23.68	9.97, 4.50-15.44, 1-44.37 ± 11.50	0.168
Time from injury to MRI, mo (mean, 95% CI, range ± SD)	12.50, 2.11-22.89, 0.07-80.33 ± 21.86	4.60, 1.28-7.91, 0.07-40.13 ± 6.98	0.150
Time from MRI to surgery, mo (mean, 95% CI, range ± SD)	6.49, 2.46-10.52, 0.4-32.13 ± 8.48	5.37, 1.61-9.14, 0.23-29.13 ± 7.92	0.715

CI = confidence interval

### Surgical Findings and Evaluation of Postoperative Outcomes

Within the nested case-control analysis, surgical reports were reviewed and interventions involving the rotator cuff tears were noted. To account for potential differences in rotator cuff assessment on preoperative imaging versus intraoperative findings, interobserver reliabilities were done. A fellowship-trained orthopaedic surgeon blinded to patient allocation conducted an independent MRI assessment of rotator cuff integrity, the results from which agreement levels were calculated. Postoperative outcomes included rate of recurrent instability, rate of return to full activity, and the postoperative VAS pain scale. To assess outcomes pertaining to military readiness, academy graduation rate, the need for a medical profile to restrict activity level, and rate of medical board evaluation resulting in separation from active duty were evaluated. Military performance outcomes and retention statistics were collected for all patients, with data retrieved from a closed healthcare system.

### Surgical Technique

All cadets underwent a shoulder stabilization procedure by one of six fellowship-trained orthopaedic sports surgeons. All cases were done in the beach chair position. For arthroscopic procedures, a posterior viewing portal is established first, followed by placement of two anterior working portals. For open procedures, diagnostic arthroscopy was done first, followed by a standard deltopectoral approach with subscapularis split or tenotomy. Significant glenoid bone loss (greater than 15% to 20%) is an indication for the Bristow-Latarjet procedure.^15-17^ Subscapularis or full-thickness supraspinatus tears were repaired, whereas small partial articular-sided supraspinatus tendon avulsion (PASTA) lesions were managed with débridement or observation. Patients underwent a standardized physical therapy protocol after surgery with 6 weeks in a sling followed by range of motion progression. Return to military duties is patient specific, but typically occurs 6 to 9 months postoperatively.

### Statistical Analysis

To estimate the incidence of rotator cuff tear in the entire cohort, we calculated the incidence proportion for rotator cuff tears along with 95% confidence intervals (CIs). The mean, SD, standard error, range, and 95% CIs were calculated for all continuous variables. Two-tailed dependent Student *t*-tests were used for analyses of continuous data between groups, whereas Chi-square or Fisher exact tests were used for categorical variables, as appropriate. Interobserver reliabilities for imaging studies in the nested analysis were calculated based on percent agreement and Cohen kappa statistics (0 to 0.2 no agreement, 0.21 to 0.39 minimal, 0.4 to 0.59 weak, 0.6 to 0.79 moderate, 0.8 to 0.9 strong, and > 0.9 almost perfect).^18^ Statistical analysis was done using STATA version 14.0 software.

## Results

### Rotator Cuff Tear Incidence

Eighteen patients (5.56%; 95% CI: 3.06% to 8.05%) had MRI findings positive for concomitant rotator cuff tears and capsulolabral injury, 17 of whom underwent an arthroscopic stabilization and were included in the matched cohort analysis (Table 3). Rotator cuff tear patterns included PASTA (6 total, of which 1 was excluded as the patient underwent open Bankart repair), partial infraspinatus tendon tear (5), partial bursal-sided supraspinatus tendon tear (3), full supraspinatus tendon tear (2), partial subscapularis tendon tear (1), and partial teres minor tear (1). Interestingly, only 4 of these 17 (23.5%) patients were reported to have evidence of rotator cuff pathology intraoperatively (Table 4). Two patients had a PASTA lesion, which correlated with MRI, one had a PASTA lesion but full supraspinatus tear on MRI, and one had a full supraspinatus tendon tear but PASTA lesion on MRI. Of the patients with rotator cuff tears, one patient underwent repair (full supraspinatus tear), two underwent débridement (PASTA lesions), and one did not have any intervention (PASTA). One patient in the no-RCT group was found to have a partial subscapularis tendon tear intraoperatively, which was repaired. Interobserver reliability between radiologist and surgeon reads of MRI studies demonstrated an agreement of 73.5% (kappa = 0.471).

**Table 3:** MRI Reports of Rotator Cuff Findings in All Patients

Variable	Category	n	%
No rotator cuff tear	—	306	94.44
Rotator cuff tear	Cumulative	18	5.56
	PASTA	6	1.85
	Bursal supraspinatus	3	0.93
	Full supraspinatus	2	0.62
	Partial infraspinatus	5	1.54
	Partial subscapularis	1	0.31
	Partial teres minor	1	0.31

PASTA = partial articular-sided supraspinatus tendon avulsion

**Table 4:** Surgical Findings and Procedures Done in Arthroscopically Managed Patients With Rotator Cuff Tear (RCT) and Without Rotator Cuff Tear (No-RCT) on MRI

Variable	Category	Rotator Cuff Tear Group (RCT)	No Rotator Cuff Tear Group (No-RCT)
Rotator cuff status on arthroscopy, n (%)	Normal	13 (76.5)	16 (94.1)
	Full tear supraspinatus	1 (5.9)	0 (0)
	PASTA	3 (17.6)	0 (0)
	Partial subscapularis	0 (0)	1 (5.9)

Procedure done	Category	Rotator Cuff Tear Group (RCT)	No Rotator Cuff Tear Group (No-RCT)
	Repair	1	1
	Débridement	2	0
	None	14	16

PASTA = partial articular-sided supraspinatus tendon avulsion

### Baseline Variable Analysis

No association was found between the presence of a rotator cuff tear and the measured baseline variables within the nested cohort analyses (Table 2). The average age of patients in the RCT and no-RCT groups was 20.76 ± 1.75 (range 18 to 24 years) and 20.18 ± 1.74 years (range 18 to 24 years), respectively (*P* = 0.373). In both groups, there were 16 males and 9 left shoulders. The mean duration of follow-up was 44.08 ± 23.34 months for both groups combined (range 6.57-84.67 months), and there were no statistically significant differences in follow-up between the RCT group (43.38 ± 23.05 months; range 6.57-81.60 months) and the no-RCT group (44.78 ± 24.31 months; range 9.83-84.67 months) (*P* = 0.878). Additional metrics, which did not demonstrate statistically significant differences between the RCT and no-RCT groups, included preoperative VAS pain scale (1.82 ± 1.98 versus 1.47 ± 2.12; *P* = 0.455), duration of preoperative physical therapy (10.35 versus 7.88 months; *P* = 0.464), average time from injury to surgery (18.99 ± 23.26 versus 9.97 ± 11.50, *P* = 0.168), average time from injury to MRI completion (12.5 ± 21.86 versus 4.60 ± 6.98, *P* = 0.150), and average time from MRI to surgery (6.49 ± 8.48 versus 5.37 ± 7.92, *P* = 0.715).

### Case-control Outcome Analysis

The presence of a rotator cuff injury on preoperative MRI did not demonstrate an association with postoperative rate of recurrent instability, activity level, or the specified military metrics. Three of the 34 patients in the nested case-control analysis experienced recurrent instability episodes (8.82%, 2 RCT versus 1 no-RCT, *P* = 0.579). Of these, none had rotator cuff tears identified on arthroscopy. One patient in the RCT group had a partial infraspinatus tear identified on MRI. He sustained a redislocation event 6 months postoperatively; however, after a subsequent course of conservative management alone, he experienced no further instability episodes. The other RCT group patient reported recurrent subjective instability during an obstacle course 8 months after surgery. He remained on active duty for another year but then underwent a medical board for a separate issue. The patient in the no-RCT group redislocated at 43 months postoperatively during a military training exercise and endorsed continued subjective instability thereafter. Return to full activity was achieved in 30 of 34 (88.24%) patients, including 15 patients (88.2%) in each group (*P* > 0.999). Medical profiles restricting activity were issued to 5 patients (14.7%), 2 of which were permanent, and 3 of which were temporary. Two patients in the RCT group and one patient in the no-RCT group required temporary profiles, one of whom remained unable to do overhead activities at final follow-up. Both permanent profiles were given to patients from the no-RCT group due to the inability to do overhead tasks, with both patients ultimately separating from the military within 5 years after surgery due to their shoulder (versus none in the no-RCT group, *P* = 0.163). A total of 30 of 34 patients (88.24%) successfully graduated from the service academy (Table 5). Of those who failed to graduate, however, none failed secondary to continued shoulder symptoms.

**Table 5:** Military-specific Outcomes Between Rotator Cuff Tear and No Rotator Cuff Tear Cohorts

Variable	Category	Rotator Cuff Tear Group (RCT)	No Rotator Cuff Tear Group (No-RCT)
Graduated from academy, n (%)		16 (94.12)	14 (82.35)
Medical profile, n (%)	Permanent	0 (0)	2 (11.76)
	Temporary	2 (11.76)	1 (5.88)
	None	15 (88.24)	14 (82.35)
Returned to full activity, n (%)		15 (88.24)	15 (88.24)
Medical evaluation board (MEB), n (%)		0 (0)	2 (11.76)

## Discussion

Several studies have examined the incidence of rotator cuff injury in the setting of glenohumeral instability in older patient populations.^8,9,19^ The present retrospective study was conducted to identify the incidence of concomitant rotator cuff tear in a consecutive series of young active-duty military personnel undergoing shoulder stabilization surgery and compared outcomes in arthroscopically managed patients with a matched cohort of patients with isolated instability pathology. Several important additional findings were noted in the current study. First and foremost, we observed an incidence of 5.88%, which is notably lower than the reported range in previous studies examining older and more heterogeneous patient populations.^20^ Interestingly, regarding MRI interpretation of rotator cuff pathology within the matched cohort analysis, the current study noted a weak level of agreement (73.5%) between radiologist and surgeon reads, as classified by kappa coefficients.^18^ Nonetheless, this finding is similar to a prior study by the Multi-center Orthopaedic Outcomes Network Shoulder group, which demonstrated an agreement of 76.7% to 78.2%.^21^ It is possible that interval healing of tears between the time of imaging and surgery may also account for the lower rate of tears identified arthroscopically, as prior studies evaluating nonsurgical management of rotator cuff tears have demonstrated decreases in tear size on follow-up MRI, particularly in patients aged <60 years.^22,23^ Nonetheless, data within young adult populations remain limited and require further investigation.

Second, no association was noted between the presence of rotator cuff tear and baseline patient variables. Although there is ample evidence regarding the evolution of nonsurgically treated rotator cuff tears in the elderly, there is a lack thereof in physically active young adult populations.^23,24,25,26^ Safran et al^27^ reported a 49% chance of tear progression in a study cohort less than 60 years of age at mean 29-month follow-up, but the youngest patient in their series was 35 years old. In addition, several studies have shown that shoulder instability events have been associated with a higher prevalence of intra-articular injury, including rotator cuff tears.^28-30^ Conversely, Orvets et al demonstrated that patients undergoing MRI greater than 6 months from the time of the initial dislocation are less likely to have evidence of a rotator cuff tear than those undergoing MRI at an earlier postinjury date (24% versus 50%, respectively).^31^ The authors attributed this finding to a significantly higher mean patient age in the group undergoing earlier MRI (39.4 versus 29.9 years) and the overall higher likelihood of rotator cuff tear in these older patients. Given the lack of corresponding data in a younger patient demographic, an analysis of time from injury to MRI was done in this study. Although a higher average time within the RCT group was noted, the difference was not statistically significant (12.50 versus 4.60 months, *P* = 0.150). Larger comparative studies in young adults are needed to draw correlations between rotator cuff tear incidence on MRI and the timing of imaging, as proposed by Orvets et al in their study.

Finally, the presence of rotator cuff tear on MRI was not associated with inferior clinical outcomes within the reported categories. In the nested, matched cohort analysis, 88.2% of patients with MRI findings of concomitant rotator cuff tear returned to full activity, whereas 11.76% of these patients experienced a recurrent instability episode. This proportion was notably higher than the 5.88% found in the no-RCT group, and although the difference was not statistically significant, it remains to be seen whether this determination would hold given a larger sample size. Voos et al^32^ reported clinical outcomes in 30 patients with combined rotator cuff and labral lesions who underwent arthroscopic repair with an average of 2.7 years of follow-up. The mean American Shoulder and Elbow Surgeons Shoulder Score score was 92.9, 90% of all patients reported good to excellent satisfaction, and 77% of patients returned to their preinjury level of athletics. Notably, no patient had a recurrent instability episode. However, it should be noted that their cohort was significantly older (average 47.8 years) than the current study group and likely subject to lower levels of occupational demand.

This study has some notable limitations. First, this is a retrospective cohort study relying on documentation of rotator cuff tear at the time of MRI and arthroscopic examination to identify incident cases, and tears not documented in the medical record may have been missed. In-season versus off-season athletic status and the scheduling of military training exercises are patient-specific factors, which affected management decisions. As a result, the timing of initial MRI from the time of injury was not standardized. Saqib et al^33^ reported a 71% sensitivity and 86% specificity of magnetic resonance arthrogram in the detection of rotator cuff tears in comparison to arthroscopic benchmark; however, in the current study, usage of contrast was not standardized, and evaluation of the rotator cuff within the subacromial space was not routinely done in all patients within the RCT group. These cumulative factors may have resulted in underreported findings and may have limited the magnitude of correlations observed between MRI findings and intraoperative observations with regard to the rotator cuff. Future prospective cohort studies with standardized imaging protocols are needed to establish more reliable estimates of rotator cuff tear incidence in this patient population. The formulation of a matched cohort analysis lends to selection bias, and a complete match of all intended variables was not possible. In addition, statistical power to detect differences in the nested analysis may have been limited, given the small sample sizes. Strengths of this study include the homogeneous characteristics of its patient population and the ability to gather military performance outcomes and retention statistics in all patients, with data collected from a closed healthcare system.

## Conclusion

Glenohumeral instability and rotator cuff injury may present concomitantly in young and physically active military service members, although the observed incidence is low. The current matched cohort analysis demonstrated no difference in baseline factors between patients presenting with and without rotator cuff tear. The presence of a rotator cuff injury on preoperative MRI does not seem to be directly associated with inferior functional outcomes or lower active-duty retention rates after surgical stabilization of the shoulder, although a higher but statistically insignificant incidence of recurrent instability was demonstrated. Larger scale prospective cohort studies with standardized imaging protocols are needed to establish more reliable estimates of rotator cuff tear incidence in young patients with acute traumatic glenohumeral instability events.
